# Non-Invasive Monitoring of Hemodialysis Patients: Challenges and Benefits in the Real World

**DOI:** 10.3390/clinpract16060098

**Published:** 2026-05-22

**Authors:** Orsolya Sáfár, Viktor Horváth, Árpád Kézdi, Péter Kevei, Ákos Géza Pethő

**Affiliations:** 1 Faculty of Medicine, Semmelweis University, 1085 Budapest, Hungary; safar.orsolya@stud.semmelweis.hu; 2 Department of Internal Medicine and Oncology, Faculty of Medicine, Semmelweis University, 1085 Budapest, Hungary; horvath.viktor@semmelweis.hu; 3 Institute of Preventive Medicine and Public Health, Semmelweis University, 1085 Budapest, Hungary; kezdi.arpad@stud.semmelweis.hu; 4 National Dialysis Center, Semmelweis University, 1085 Budapest, Hungary; peter.kevei@nemzetidializis.hu

**Keywords:** hemodialysis, cardiovascular mortality, sudden cardiac death, electrolyte changes, QT interval, Holter ECG, patient compliance

## Abstract

**Background**: Cardiovascular complications are the leading cause of death in patients with end-stage renal disease (ESRD). Hemodialysis involves rapid electrolyte shifts and sudden fluid removal, which can affect ventricular repolarization and trigger arrhythmias in patients with ESRD. To enhance patient care, it is crucial to regularly assess cardiac function using noninvasive and painless methods, such as Holter electrocardiography (ECG) and routine cardiac ultrasound. These evaluations may inform improved prevention strategies to reduce the risk of elevated cardiovascular mortality rates. **Methods**: In total, 40 patients with ESRD on chronic hemodialysis (HD) were approached, and only 18 were enrolled from September 2024 to July 2025. Detailed medical information was provided, and written informed consent was obtained from the patient. The median duration of Holter ECG recording was 84.65 h, and cardiac ultrasound examinations were conducted. Blood gas samples were collected hourly during the second dialysis session. **Results**: Surprisingly, one-third of the patients opted to withdraw their consent for this painless investigation. No significant differences were observed in the QT and QTc intervals between the dialysis and non-dialysis days (*p* = 0.184 and *p* = 0.446, respectively). However, a significant increase was observed during the first 3 h of dialysis when analyzing the intradialytic period. **Conclusion**: Some patients showed clinically significant changes in QT and QTc intervals during treatment, which could not be confirmed statistically. Although we did not formulate a hypothesis, it is essential to recognize that patient compliance significantly influences the cardiovascular outcomes of individuals undergoing hemodialysis.

## 1. Introduction

Individuals with chronic kidney disease (CKD) have a significantly higher mortality rate than the general population. Worldwide, approximately 850 million people are affected by CKD, and more than 2.7 million receive hemodialysis (HD) treatment, which is associated with an even greater risk of mortality [1,2]. According to data from the United States Renal Data System (USRDS) in 2020, for which the cause of death was documented, cardiovascular disease was responsible for 51.8% of all cases. Among these, arrhythmia/cardiac arrest was the leading cause in 41.8% of all cases. In addition to these factors, withdrawal from dialysis and various infections (such as COVID-19) also play a significant role in mortality [3]. Among patients who receive HD treatment three times per week, sudden cardiac death (SCD) is most likely to occur within the last 12 h of the dialysis-free weekend or within the first 12 h after the beginning of the HD session [4,5]. There are several differences between HD treatment and normal kidney function that could explain the increased incidence of SCDs, including sudden fluid removal, rapid electrolyte and pH changes, and myocardial fibrosis caused by uremic toxins [6,7]. One of the underlying causes of SCD is prolonged QT and QTc intervals [8,9,10,11]. However, the effects of HD on ECG parameters remain unclear. Some studies have measured ECGs both before and near the end of the HD session and reported QTc interval prolongation [12]. Conversely, in other studies, increased QT dispersion was associated with a significantly shorter QTc interval immediately after HD treatment [13]. Based on our hypothesis, HD-treated patients are at an increased risk of developing cardiac arrhythmias due to the constantly changing ion and acid–base homeostasis. To enhance patient care, it is essential to conduct regular assessments of cardiac function using non-invasive and painless methods, such as Holter electrocardiography and routine cardiac ultrasound. These measures may contribute to improved prevention strategies aimed at reducing the risk of increased cardiovascular mortality. The goal of our study was to monitor our patients over an extended period (96 h) using a Holter ECG and to monitor electrolyte and pH fluctuations during HD treatment through blood gas analysis. This approach enabled us to continuously monitor changes in QT and QTc intervals during and between HD sessions and to investigate potential correlations with changes in ion levels, particularly potassium. To the best of our knowledge, this is the first study to examine these parameters over a long period.

## 2. Materials and Methods

### 2.1. Ethical Issues

This study was approved by the Hungarian Medical Research Council (ETT TUKEB BM/13024-1/2024). This study adhered to the Declaration of Helsinki established by the World Medical Association. Routine hemodialysis prescriptions remained unchanged; no additional interventions were performed; and participants were approached consecutively. The patients enrolled in our study signed an informed consent form after receiving detailed information about the study. All patients underwent hemodialysis at Semmelweis University, Budapest, Hungary. The capacity of the hemodialysis center and the number of treated patients determined the number of participants included in our study.

### 2.2. Data Collection

This study was conducted at the Dialysis Unit of the Department of Internal Medicine and Oncology at Semmelweis University. All patients underwent HD three times per week. Holter ECG monitoring was initiated on the first dialysis day of the week and was discontinued on the third day of dialysis. Given our available capacity, we successfully included patients who received HD on Mondays, Wednesdays, and Fridays in our study. On the first day of the week, a cardiology expert performed transthoracic echocardiography. Subsequently, we equipped the patients with programmed Holter-ECG devices (Labtech, Debrecen, Hungary). Given the extended duration of Holter-ECG recordings, we also took the time to educate patients about the importance of replacing the batteries. Changes in serum ion concentration and pH were monitored during the second dialysis session using hourly arterial blood gas analysis.

The median duration of Holter ECG recordings was 84.65 h, and the recordings were performed using an EC-12H Recorder (Labtech, Debrecen, Hungary). Each recording included the entire duration of hemodialysis and the following non-dialysis day, which comprised a morning resting period that served as a comparison window. This criterion served as the primary basis for selecting records. Shorter monitoring durations were due to patients’ failure to replace the batteries as scheduled. We used a 12-lead configuration, and the device recorded and stored each patient’s heartbeat until it reached its data storage limit. Holter-ECG recordings for each patient underwent meticulous manual review to eliminate any noise. Subsequently, the refined noise-free recordings were subjected to automated analysis using specialized software. The resulting data were validated by a cardiologist who contributed to the study. QT intervals were measured automatically, and heart rate correction was performed using Bazett’s formula [14,15].

### 2.3. Echocardiographic Investigations

Transthoracic echocardiography was performed by a cardiology specialist on the day of the first dialysis, either immediately before or after treatment. The Vivid S70N ultrasound system (GE Healthcare, Chicago, IL, USA) was used for echocardiographic imaging, including two-dimensional, M-mode, and Doppler imaging. Standard two-dimensional echocardiographic projections were used to assess the left heart structures (ascending aorta, aortic root, left atrium, left ventricle, interventricular septum, and posterior wall of the left ventricle) and to evaluate the right side by measuring the right atrium, TAPSE (M-mode), and inferior vena cava diameter. The Simpson’s biplane rule was used to calculate the ejection fraction. Pulsed-wave and tissue Doppler echocardiography were used to characterize left ventricular diastolic function, including E and A wave velocities, E/A ratio, deceleration time (DT), and movement of the mitral annulus (Ea, Aa, and Sa waves). To assess the systolic output, LVOT-VTI, LVOT-vmax, and Ao.vmax were recorded.

### 2.4. Hemodialysis Prescription

We included patients with arteriovenous fistulas and those with hemodialysis central venous catheters treated with Fresenius 5008S devices (Fresenius Medical Care, Bad Homburg, Germany) in this study. The patients underwent four-hour high-flux bicarbonate hemodialysis using a body weight-dependent polyethersulfone membrane surface (Nipro ELISIO™ 17 or 21; Nipro Corporation, Mechelen, Belgium). The type of vascular access did not influence the results of this study. Among the HD settings, bicarbonate was adjusted based on the arterial blood gas results for all patients, thereby preventing acid–base imbalance at the end of HD. In accordance with the local protocol, all patients were treated uniformly with a 3 mmol/L potassium-containing concentrate. The common settings were as follows: Na^+^, 138 mmol/L; K^+^, 3 mmol/L; Ca^2+^, 1.25 mmol/L; bicarbonate, 30 mmol/L.

### 2.5. Laboratory Assays

During the HD sessions, blood samples (2 mL each) were collected from the arterial line of the dialysis circuit every hour for a total of five times. The concentrations of K^+^, Na^+^, Ca^2+^, Cl^−^, HCO^3−^, and the base excess were analyzed using an ABL90 FLEX PLUS blood gas analyzer (Radiometer Medical ApS, Copenhagen, Denmark). In our study, we did not collect patients’ monthly laboratory results because they represent a single point in time and do not reflect changes that occur during HD.

### 2.6. Data Analysis

Descriptive data are presented as counts and percentages for categorical variables and as mean ± standard deviation or median (interquartile range) for continuous variables. The QT and QTc intervals were evaluated at nine predefined time points (0, 30, 60, 90, 120, 150, 180, 210, and 240 min), constituting repeated measurements within the same subjects. Normality was assessed using normality tests and visual inspection of histograms and Q-Q plots. Non-normally distributed variables were logarithmically transformed as necessary. To compare the clinical characteristics across groups, normally distributed continuous variables were reported as mean ± standard deviation and analyzed using a *t*-test. Categorical variables were compared using Fisher’s exact test. To evaluate the overall differences in QT/QTc values across time points, repeated-measures analysis of variance (RM-ANOVA) was used. Given the modest sample size and potential deviations from normality, a non-parametric Friedman test was also conducted as a sensitivity analysis. The effect size for the Friedman test was quantified using Kendall’s W. To formally assess temporal trends in QT/QTc duration, a linear mixed-effects model was fitted with time (in minutes) as a fixed effect and patient identifier as a random intercept, thereby accounting for within-subject correlation in repeated observations. Model estimates are presented as regression coefficients (β) with corresponding 95% confidence intervals (CIs). To investigate potential non-linear patterns, an additional model incorporating a quadratic time term was assessed and compared with the linear model using a likelihood ratio test. Additionally, the QT/QTc values were averaged at each time point, followed by a variance-weighted least squares (WLS) regression on these means to estimate the overall temporal trend while accounting for the heteroscedasticity of the mean estimates. Statistical significance was set at *p* < 0.05. Statistical analyses were conducted using SPSS version 29.0.1, and figures were created using GraphPad Prism 11 software (GraphPad Software, La Jolla, CA, USA).

## 3. Results

### 3.1. Patient Characteristics

**Figure 1:**
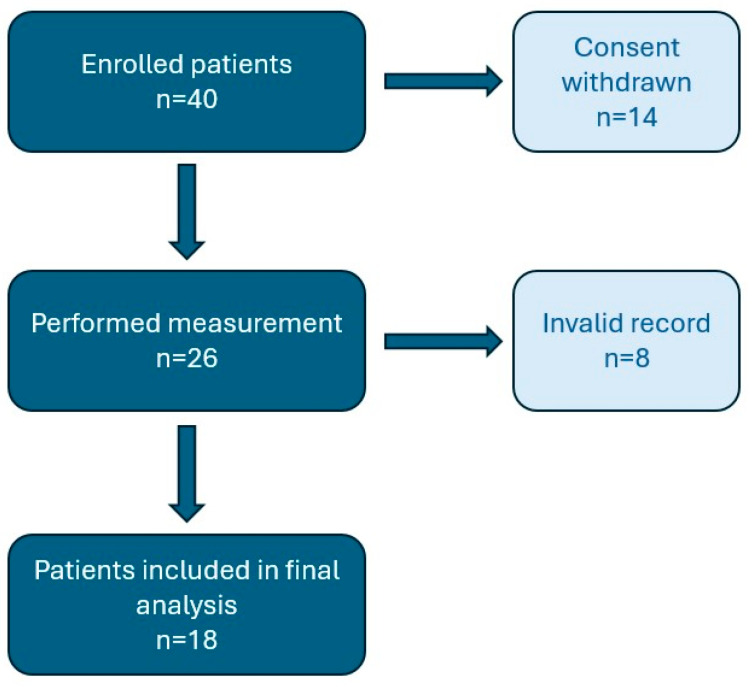
Flowchart of patients included in the study. We included a total of 40 patients in our study; however, we ultimately included only 18 patients in the statistical analysis.

The number of patients eligible for inclusion in our study was determined by the dialysis station’s capacity and the availability of cardiology appointments. Additionally, the feasibility of conducting cardiac ultrasound examinations and programming the Holter-ECG device imposed further constraints. Ultimately, a total of 40 patients undergoing hemodialysis were included in our study. Patient selection was random, adhering to the limitations. Each patient received thorough information about the study and subsequently provided their written consent. Fourteen withdrew their consent before the echocardiography, and in eight cases, the Holter-ECG record was invalid. The invalid ECG recording was caused by patient non-compliance; the batteries were not replaced on time, or the patient removed the electrodes too early. The final analysis included 18 participants (Figure 1). Patients who ultimately declined the test did not provide a clear rationale for their decision. The most frequent response was that they had reconsidered and no longer wished to undergo this painless, non-invasive procedure. Given the voluntary nature of the study, we did not explore further reasons for refusal. The data obtained from each patient passed the normality tests for a Gaussian distribution using the D’Agostino–Pearson omnibus test, the Shapiro test, and the Kolmogorov–Smirnov test.

There were equal numbers of male and female patients (n = 9; 50% each), with a mean age of 65.1 years for men and 59.1 years for women (Student’s *t*-test, *p* = 0.373). The primary underlying cause of chronic kidney disease was diabetic nephropathy (n = 8, 44.4%). (Table 1) The average duration of HD was 35.8 months for men and 36.5 months for women (Student’s *t*-test, *p* = 0.963). The baseline characteristics are presented in Table 2.

### 3.2. Laboratory Results During Hemodialysis

By analyzing blood gas samples during hemodialysis, we observed, using statistical analysis, significant increases in pH and serum bicarbonate levels and a significant decrease in serum potassium concentration (ANOVA, *p* < 0.05), consistent with the effectiveness of the dialysis treatment. In contrast, no significant changes were detected in serum sodium and calcium levels (Table 3).

### 3.3. Echocardiographic Investigations Results

All patients underwent transthoracic echocardiography on the day of their initial dialysis session, either immediately before or after the treatment. The primary aim of this examination was to exclude any signs of severe heart failure or critical cardiac conditions from the study. Additionally, we sought to identify significant wall motion abnormalities or notable left ventricular hypertrophy that could affect Holter-ECG findings. Based on the echocardiographic findings from patients in our study, we observed surprisingly good left ventricular systolic function. The echocardiographic results are summarized in Table 4.

### 3.4. Holter-ECG Results

**Figure 2:**
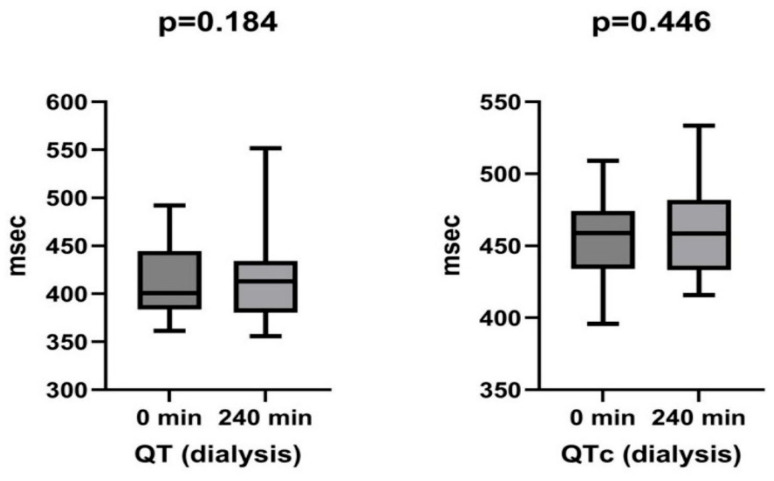
Comparison of QT and QTc intervals at the beginning and end of hemodialysis. We observed increases in both parameters, but no significant differences were found.

The median Holter ECG recording time was 84.65 h (planned 96 h), with a median of 340,114 cardiac cycles recorded. In our analysis of Holter ECG data, we tested our study hypothesis and assessed arrhythmia occurrence among patients. Ultimately, six patients exhibited supraventricular tachycardia, while three experienced non-sustained ventricular tachycardia. No other pathological arrhythmia was documented in our patients. Consistent with previous studies, we initially compared the mean QT and QTc intervals at the onset (0 min) and conclusion (240 min) of hemodialysis. However, unlike earlier studies, we used averages of 5 min at both the beginning and end of the procedure rather than a single QT or QTc interval. We observed increases in both parameters; however, statistical analysis revealed no significant differences (*p* = 0.184 and *p* = 0.446, respectively) (Figure 2).

The study analyzed changes in QTc intervals during dialysis using repeated-measures analysis of variance (RM-ANOVA), which showed no significant effect of time on the QTc values (F8, 136 = 0.88, *p* = 0.54). Similarly, the Friedman test revealed no significant differences across time points (χ^2^ = 8.87, *p* = 0.35) and a small effect size (Kendall’s W ≈ 0.06), indicating comparable QTc distributions during the observation period. A linear mixed-effects model identified a significant positive association between time and QTc duration (β = 0.026 ms/min, *p* = 0.024), indicating a mean QTc prolongation of approximately 6 ms in 240 min. The inclusion of a quadratic term did not improve the model (*p* = 0.56), indicating a linear trend. Variance-weighted least-squares regression corroborated these findings, yielding a slope of 0.0247 ms/min (*p* = 0.005), confirming a consistent upward trend in mean QTc over time.

**Figure 3:**
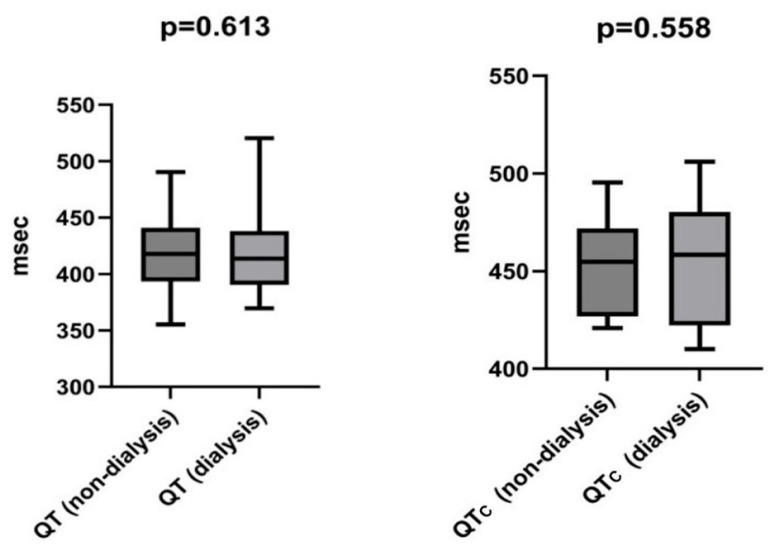
Comparison of QT and QTc intervals between dialysis and non-dialysis days. No significant differences were found in QT and QTc intervals between the two periods.

In the subsequent phase of our statistical analysis, we compared the Holter-ECG results recorded during the dialysis-free period with those obtained during a 4-h hemodialysis (HD) treatment. The Holter-ECG data collected during HD treatment were juxtaposed with the results obtained in the early morning hours (between 2 and 6 a.m.) of the dialysis-free day. This control period was selected because during the 4-h HD session, our patients at the dialysis center remained at rest, refrained from physical activity, and slept predominantly. Similarly, during the early morning hours of the control HD-free period, our patients were asleep and did not engage in physical activity. Any increase in heart rate from physical activity could have affected the QT and QTc results. Statistical analysis indicated no significant differences in the QT and QTc intervals between the two periods (ANOVA, *p* = 0.613 and *p* = 0.558, respectively) (Figure 3).

## 4. Discussion

Sudden death is one of the more frequent causes of death among HD patients, but the underlying mechanisms, the contribution of arrhythmia, and associations with serum chemistries or the dialysis procedure are not completely understood [16]. In a previously published Holter-ECG study investigating the development of malignant cardiac arrhythmia, a sudden increase in heart rate was observed, which was associated with transient ventricular tachycardia during or after hemodialysis.

In our prospective observational study, we continuously monitored ECG parameters for a median of 84.65 h, both during and between hemodialysis treatments. As observed in previous studies, the reported ECG parameters have shown inconsistent values, and QTc interval prolongation and shortening have also been observed; however, these investigations were based on occasional ECG measurements [13,17]. The 2005 ICH E14 guideline indicates that a QTc interval exceeding 10 ms is a cause for concern and requires additional monitoring of patients. A QTc greater than 20 ms is regarded as a positive result, while an individual QTc increase of >60 ms is classified as a “significant” outlier, which could potentially lead to further cardiac complications [18].

Although our goal was to cover a much longer period, we were unable to demonstrate consistent changes in any direction, and our analysis did not yield concordant results when comparing the beginning and end of the dialysis. At the same time, focusing on the intradialytic periods, relevant alterations in the heart’s conduction system presumably occur within the initial hours of HD. This may be explained by the fact that electrolytes, such as potassium, are corrected to the greatest extent at the beginning of dialysis [19]. Numerous studies have examined alterations in electrolyte levels during hemodialysis and identified them as significant risk factors for cardiovascular mortality [20,21,22]. Such abrupt changes, especially a decline in serum potassium levels, could be key factors in the instability of ventricular repolarization and contribute to the risk of arrhythmia [23]. The QTc interval is prolonged in HD patients. This phenomenon is partly due to fluid overload and fluctuations in serum magnesium and calcium levels during the initial two hours [24]. In our study, increased QT and QTc interval values were observed during the first three hours of dialysis. Earlier reports have indicated an increased incidence of sudden cardiac death on dialysis days and have observed it more frequently during the first hours of dialysis [25]. Research indicates that potassium is crucial for the development of cardiac arrhythmias in patients undergoing HD treatment [23,26].

Patients with chronic renal failure often display ECG changes and a high incidence of both ventricular and supraventricular arrhythmia, which can be significant during and after hemodialysis [27,28,29]. Although our study did not conclusively demonstrate all significant changes across the entire patient population, an independent analysis of patient data confirmed our findings. Notably, independent ECG changes were typically observed during the initial hours of the hemodialysis session, consistent with earlier reports. We hypothesize that rapid fluctuations in electrolyte levels and blood pH during hemodialysis influence the generation and conduction of cardiac impulses. Patient compliance during hemodialysis is often suboptimal, which may have significantly affected the outcomes of our study [30]. Patient compliance was not included in the hypothesis generation of this study. We were surprised by the number of participants who withdrew their consent during the study. Given that a significant proportion of patients (approximately one-third) opted out of the clinical study, we believe it is important to briefly address this issue. Those who chose to withdraw did not provide specific reasons for their decisions. The most common response was that they had reconsidered and no longer wished to undergo this painless, noninvasive procedure. Given the voluntary nature of the study, we opted not to explore further the reasons for their refusal. Consequently, it is not surprising that a notable proportion of participants in our study ultimately declined a painless and beneficial diagnostic test [3]. Research indicates that reducing or omitting at least one HD treatment per month by at least 10 min can increase mortality rates by 25–30% [31]. Our study demonstrated that patient compliance significantly impacted outcomes. Regular cardiological monitoring of patients with ESRD, who are particularly vulnerable, enables the timely recognition of worsening cardiovascular conditions.

These results, in line with our findings, suggest the need for closer observation of high-risk patients during dialysis, such as Holter ECG monitoring. This monitoring method can detect subtle changes during hemodialysis that traditional methods may miss. Although previous studies have reported an increased number of sudden cardiac deaths on dialysis days, the present analysis did not reveal any discrepancies between days [25]. This may be due to the small number of patients included in the study. Overall, our results emphasize the importance of the early intradialytic phase of treatment and indicate the need to expand the measurements in a multicenter study with larger sample sizes.

## 5. Conclusions

Hemodialysis is generally considered a safe procedure; we did not observe any malignant arrhythmias, such as sustained ventricular tachycardia, ventricular fibrillation, asystole, or third-degree atrioventricular block. However, patients undergoing chronic hemodialysis face an increased risk of cardiovascular complications, mainly due to the prolongation of the QT and QTc intervals that can occur during the procedure. Rapid changes in electrolyte levels during hemodialysis can influence cardiac impulse generation and conduction. While the findings should be interpreted with caution, given the relatively small sample size, it is noteworthy that some patients exhibited clinically significant alterations in the QT and QTc intervals over the course of treatment. However, these changes could not be statistically validated. Continuous Holter ECG monitoring in patients with ESRD could provide valuable insights, especially when other cardiovascular risk factors are present. The most important conclusion of our study is that, despite extensive efforts, the cardiovascular outcomes of patients with ESRD are primarily affected by compliance.

## 6. Limitations

The extended monitoring period and the significant amount of ECG data collected from each patient were the key strengths of our study. To the best of our knowledge, this is the first study to analyze ECG parameters using this methodology. This study had several limitations that warrant discussion. Firstly, the relatively small sample size should be noted, as it necessitates cautious interpretation of our results. This limitation arose from insufficient cooperation among patients, and future examinations of this group may yield more insightful findings. Additionally, we did not investigate potential drug interactions, and we did not measure patients’ serum magnesium levels. Our current study also lacked a comparison of Holter ECG results with interdialytic weight gain and ultrafiltration rates during hemodialysis treatment. These limitations may serve as a foundation for generating hypotheses for future research. Furthermore, patient heterogeneity, such as differences in vascular access types and underlying causes of kidney disease, may pose challenges.

## Figures and Tables

**Table 1:** Underlying causes of chronic kidney disease. Not surprisingly, based on epidemiological data, most patients suffered from diabetes.

Cause of End-Stage Renal Disease	Total (n = 18)	Frequency (%)
Diabetic nephropathy	8	44.4%
Hypertensive nephropathy	3	16.7%
Polycystic kidney disease	3	16.7%
Focal segmental glomerulosclerosis	1	5.6%
IgA-nephropathy	1	5.6%
p-ANCA vasculitis	1	5.6%
Analgesic nephropathy	1	5.6%

Abbreviations: IgA, immunoglobulin A; p-ANCA, perinuclear anti-neutrophil cytoplasmic antibody.

**Table 2:** Baseline characteristics of enrolled patients. There were no significant differences between men and women in age, time on dialysis, hypertension, or diabetes, as determined by *t*-tests and chi-square tests.

Patients’ Characteristics	Male (n = 9)	Male SD/IQR	Female (n = 9)	Female SD/IQR	t-Statistic	Chi-Square Statistic
Age (years)	65.1	13.5/24	59.1	14.3/23	*p* = 0.373	
Time on dialysis in months	35.8	28.4/43	36.5	34.3/68	*p* = 0.963	
Diabetes mellitus	5		4			*p* = 1
Hypertension	9		9			*p* = 1

Abbreviations: IQR: interquartile range; SD: standard deviation.

**Table 3:** Serum electrolyte levels and pH during the hemodialysis session. Statistical analysis reveals significant increases in pH and serum bicarbonate levels and a significant decrease in serum potassium concentration (*p* < 0.05), confirming the effectiveness of dialysis.

Laboratory Parameters Monitored During HD Sessions	In the 0th Minute of HD Treatment	SD/IQR	In the 60th Minute of HD Treatment	SD/IQR	In the 120th Minute of HD Treatment	SD/IQR	In the 180th Minute of HD Treatment	SD/IQR	In the 240th Minute of HD Treatment	SD /IQR	Repeated Measures ANOVA
pH	7.34	0.047/0.07	7.37	0.041/0.05	7.38	0.038/0.05	7.38	0.043/0.14	7.39	0.041/0.06	*p* < 0.001
SeHCO^3−^ (mmol/L)	20.71	2.26/3.3	22.09	1.94/2.7	22.62	1.87/2.3	23.43	3.34/3.1	22.96	1.56/2.7	*p* < 0.001
SeNa^+^ (mmol/L)	140.6	4.2/4.5	140.9	3.3/4	141.1	2.3/2	141.2	2.5/1	141.3	2.2/3	*p* = 0.535
SeK^+^ (mmol/L)	4.25	0.79/0.53	3.82	0.61/0.5	3.54	0.46/0.5	3.39	0.37/0.5	3.38	0.38/0.5	*p* < 0.001
SeCa^2+^ (mmol/L)	0.82	0.14/0.17	0.82	0.12/0.16	0.81	0.14/0.21	0.79	0.13/0.2	0.81	0.14/0.18	*p* = 0.906

Abbreviations: HD, hemodialysis; SeCa^2+^, serum calcium; SeNa^+^, serum sodium; SeK^+^, serum potassium; SeHCO^3−^, serum bicarbonate; SD, standard deviation; IQR, interquartile range.

**Table 4:** Echocardiography assessment indicated preserved left ventricular systolic function, with a mean ejection fraction of 59.11%. The average left ventricular posterior wall thickness was 10.72 mm, demonstrating a normal–mildly elevated wall thickness. Regional wall motion abnormality was detected in 11.11% of patients.

Echocardiography Parameters	Mean	SD/IQR	Frequency (%)
EF (%)	59.11	5.779/7	
LVPW (mm)	10.72	1.179/2	
RWMA			11.11

Abbreviations: EF: Ejection Fraction; LVPW: Left Ventricular Posterior Wall; RWMA: Regional Wall Motion Abnormality.

## Data Availability

The datasets generated and analyzed in this study are not publicly available owing to intellectual property concerns; however, they can be accessed by the corresponding author upon reasonable request.

## Abbreviations

The following abbreviations are used in this manuscript:

CKD	chronic kidney disease
ECG	electrocardiographic
EF	ejection fraction
ESRD	end-stage renal disease
HD	hemodialysis
IgA	immunoglobulin A;
IQR	interquartile range
LVPW	Left Ventricular Posterior Wall
p-ANCA	perinuclear anti-neutrophil cytoplasmic antibody.
RWMA	Regional Wall Motion Abnormality
SCD	sudden cardiac death
SD	standard deviation
SeCa	serum calcium
SeHCO	serum bicarbonate
SeK	serum potassium
SeNa	serum sodium
USRDS	States Renal Data System
